# Popular health guides and their reception in Finland, 1890s–1970s

**DOI:** 10.1017/mdh.2025.10019

**Published:** 2025-07

**Authors:** Ilari Virtanen, Kalle Kananoja

**Affiliations:** University of Oulu, Oulu, Finland

**Keywords:** Finland, medical popularisation, health guides, hygienism, irregular medicine, patients

## Abstract

This article explores the cultivation of medical knowledge via popular health guides among the Finnish lay populace from the 1890s to the 1970s. By using written reminiscences and newspaper articles as source material, the article discusses the relevance, popularity, and practical use of various printed health guides and manuals throughout Finland. We place particular focus on the late nineteenth to the early twentieth century as the period that experienced a high increase in lay education and literacy. By focusing on individual readers and their experiences of popular health guides, the article examines lay medical and health practices as the number of medical manuals dramatically increased from the late nineteenth century onwards. It also investigates the reception of medical, popular and irregular health movements, such as hygienism, nature cure, and Couéist autosuggestion, and the change in medical culture brought about by the appearance of patent medicines. As the information discovered in popular health guides tended to fluctuate between official and irregular medical theory, we analyse the relationship between learned, alternative, and vernacular medicine through the views and opinions expressed by people who engaged with health literature. Through these materials, we provide a novel understanding of the accessibility of medical knowledge, the spread and impact of health guides, and attitudes towards different medical practices among the Finnish reading public.

## Introduction

Then, at 22 years of age, began a constant throat ache. I always went to a doctor and believed it was the highest wisdom in the healing of men. But no help was forthcoming. Doctor said it is chronic. Tonsils should be taken out because they are so big. It could lead to joint and heart disease.

8 years later came all those defects, joints aching and in fever. Heart beat, beat, and stopped. It was 1931 April 15.

At this point, what ‘luck’ would have flown a man, who sold a book on the street: Rheumatism and heart disease (auth. Vainikainen).

My brother, a couple years younger than me did not care to buy it, but the man said: ‘Go ahead and buy, it’s such a good book and only costs 10 p.’

Then he came by our house and said: ‘I don’t care to carry it around, read if you like.’

I read it twice and so began cold swiping the stomach and towelling each morning. And hot water I drank to keep warm.

There were no bathing houses in the 1930s, not many in any case, but I swiped myself over a wash basin. I got up 15 minutes earlier than others, so that I had a chance to do it. The book also said, away with meat, all poisons coffee etc. medicines. Clean, light vegetarian food only.^1^

Thus commenced the narrative of a Finnish housewife, born in 1901 and residing in Riihimäki, detailing her over forty-year engagement with natural remedies, water cures, and raw food. A chance encounter on the streets of Kotka landed a nature cure tract in her hands. Having become popular in Finland around 1910, nature cure was on the wane by the 1930s but V. Vainikainen nevertheless propagated the idea and its methods.^2^ Published in Viipuri, South Karelia, where nature cure had taken root two decades earlier,^3^ his book was relatively well received and gained a few favourable reviews in newspapers.^4^ Although he published another title on the same topic,^5^ Vainikainen did not become a well-known author. How could such a seemingly insignificant booklet, then, exert such a profound impact in someone’s health behaviour?

Vainikainen’s small booklet was just one of numerous health guides published in Finland between the 1890s and 1970s. These publications can be divided roughly into two categories. First were the practical domestic medical guides written by medical doctors, both translations and original works by Finnish physicians.^6^ The most popular books tended to have a long lifespan and were usually reprinted several times. By the 1900s, as medical popularising became more common in Finland, guidebooks had transformed into larger volumes often co-written by several physicians. While translations remained important, the prominence of Finnish-authored books increased. Second, from the 1890s, the book market was increasingly saturated by irregular^7^ health manuals authored by laypeople with little or no official medical training. Germany was the most important origin of irregular medical practices imported to Finland, while migrants returning from the United States also contributed new ideas.^8^ Similar to popular medical books authored by doctors, Finnish non-physician authors soon began to publish a variety of health guides, as Vainikainen’s example above demonstrates.

This article analyses popular health guides and their reception in Finland between the 1890s and 1970s, with a particular focus on twentieth-century experiences. While it includes medical books as source material, the article does not engage in a detailed close reading of these books. Instead, it examines Finnish physicians who interacted with the lay audience by providing commentary on medical books in newspapers and periodicals. Our research is also based on written reminiscences by laypeople, and we especially want to highlight a 1973 enquiry on folk medicine undertaken by the Finnish Academy of Science and Letters and the Department of Folklore Studies at the University of Turku. This material gives us a rather unique opportunity to study the readers of popular health guides, and our approach departs from earlier studies that have maintained that it is impossible to say whether readers made use of these manuals.^9^

This study is especially concerned with the usage of medical books. What kind of knowledge did this literature provide to readers, and how did it evolve? What meanings did their readers give them, and how did they put into practice what they learned through the written word? To tackle these questions, the article draws upon the 1973 survey regarding folk and popular medicine, from which the opening quote is also drawn. We argue that while the hygienistic literature composed by physicians provided the population with important guidelines on healthy living, the ‘miracle cures’ advocated by irregular healers and patent medicines marketed via print by pharmacists had a wide popular appeal. Further, we show that, in some instances, the gap between officially approved ‘scientific’ practices and irregular medical knowledge was not remarkably wide.

The article first examines the development of medical popularisation and its effect on public perceptions of health by providing an overview of the historiography concerning the role of laypersons in the dissemination and reception of medical knowledge. The section on budding health literature traces the emergence and development of Finnish-language medical and health literature, highlighting early key publications (including translations) and their impact. We also discuss the reception and use of health manuals authored by Finnish physicians, considering their influence on public health practices and attitudes, before turning to the subgenre of sexual health guides, examining their content and the role they played in educating women and men about intimate health. The final section delves into various health fads and irregular healing methods that played a role in shaping lay practices and attitudes among the Finnish reading public.

## Earlier research and sources on lay readership

The development of medical popularisation and its effect on lay perceptions of health has been studied in a variety of historical settings, but with a strong emphasis on the early modern period. Although ‘western’ biomedicine has dominated these narratives, the issue has also been raised in a wider global context.^10^ As Roy Porter has argued, the time- and culture-specific genre of medical popularisation depended upon a number of preconditions, including the facilities for the widespread production and distribution of books, education and literacy, and an ethic inculcating self-help. On a practical level, the necessity of educating the public about perceived health threats was coupled with a shortage of physicians and adequate health care. Popular health guides had the potential to transform health culture, but, as Porter remarked, we do not know how far owners actually read the books in their possession.^11^ By focusing on readers, we aim to assess not only ‘the patient’s view’, but also to demonstrate that Michael Worboys’ concept of non-patients – those people who never consulted a doctor but instead sought information from friends, family, and books – holds limited relevance within the Finnish context.^12^ People often turned to medical manuals in addition to, not instead of, seeking a doctor’s opinion.

In her pioneering work, ethnologist Anna-Maria Reinilä has charted the use of medical literature and health guides among laypersons in Finland by studying estate inventories and private library catalogues. The primary source critical challenge identified in her material is the discrepancy between the ownership of books in private libraries and their actual utilisation. Despite being listed in an individual’s private collection, there is no guarantee that the owner has engaged with the content or implemented the recommended practices. Focusing mostly on the early modern period and the nineteenth century, her definition of laymen included barbers, clergymen and parish sextons, who had a semi-official position as healthcare providers and bloodletters.^13^

While acknowledging the semi-official dimension of healthcare explored by Reinilä, we want to expand the definition of laypersons. As Nieto-Galan has noted, ‘the idea that there existed well-defined groups of experts and laypeople is oversimplified. The so-called amateurs […] and the supposed laypeople are all dynamic and changing agents […]’.^14^ In our analysis of readers of medical literature, then, we include all individuals, regardless of social position.

Throughout the 19th century, Finnish literacy was still relatively undeveloped compared with many other European regions. Although in 1880 only 2.4 per cent of the population over the age of ten was considered illiterate, by contrast, only 4.6 per cent were able to write. Popular education was still, for a large part of the century, in the hands of the clergy, who understood literacy as a supplement to religious practice. There existed two socially defined types of literacy: the modern type of the educated and the upper classes, and ecclesiastical literacy, which meant being able to ‘read’ Luther’s *Small Catechism* and other books that explained or interpreted it.^15^

Clerical-led education did not cultivate any type of active literacy but focused on the ability to recite key passages from religious sources. This gradually began to change from the 1860s onwards with the rise of Finnish-language newspapers and the founding of the first official public schools.^16^ Like other western countries, Finland underwent a transformation that made new groups of readers – women, children and workers – part of the reading public.^17^ However, as Kati Mikkola has shown, religious literature remained the mainstay of book ownership among the peasantry even in the early twentieth century, especially the further away one moved from urban centres to the peripheries.^18^

To understand the reception of health manuals among readers, we studied the 1973 folk medicine survey, reposited at the Archives of History, Culture and Arts Studies at the University of Turku, which consists of responses from 404 individuals written on over 10,000 lined cards. The research design was simple. The respondents were sent a questionnaire, which included thirty-eight questions regarding folk medical practices and healing. Only one set of questions focused specifically on medical literature and health, namely: ‘35. Has there been a medical book [*lääkärinkirja*, literally doctor’s book] at home? Has it been used? Name of the book and author?’^19^

The answering techniques of the respondents varied. Some answered mechanically to the questions in numerical order and neatly numbered their answers, while others provided unstructured and jumbled narratives. Most answers concerned healers, healing techniques and home remedies. Eighty respondents (twenty per cent) included information on books. Some answered directly that they had never encountered published health guides in domestic settings.

Chronologically, the answers included information from the eighteenth century up to the early 1970s, or the very recent past for the respondents. Most answers, however, narrated events that had taken place in the first half of the twentieth century.^20^ Geographically, answers came from all parts of Finland, including Lapland, but the majority of information was provided by respondents who lived south of the Vaasa-Kuopio-Joensuu line.

Besides published health guides, literate autodidact folk healers were another recurring theme in the written reminiscences.^21^ In the University of Turku corpus, many informants revealed memories of literate healers, who sought medical knowledge from books, newspapers and magazines, but also from medical doctors and other learned people and healers. There were also memories of priests, whose training had probably often included medical skills and knowledge.^22^ In addition to the survey conducted by the folklorists at the University of Turku, the Finnish Literature Society collected information on folk healing in 1977–1978. It is notable, but not surprising, that autodidaction still played some role when ailing individuals sought ways to health after 1950, as revealed by the letters in the Finnish Literature Society’s collection. For example, one informant wrote about methods she had learned in Swiss phytotherapist Alfred Vogel’s book *Luonto paras lääkitsijä* (*Nature Best Medicine*) and Teo Snellman’s *Parantava paasto* (*The Healing Fast*).^23^

The written reminiscences studied in this article have their own deficits and biases. The informants often referred to incidents that had taken place several decades earlier, and their anecdotal memories were sometimes hazy and imprecise. For example, book titles could have been forgotten or were recalled imprecisely, making it challenging to identify the exact publication in question. However, because books were tangible objects, they were sometimes passed in the family over generations, making identification certain. As the opening quote alludes, some informants could have a very personal and deep relationship with health guides, and their testimonies open up possibilities for ‘thick description’.^24^ What is noteworthy, however, is that many informants recalled books focusing on irregular medical practices. A possible explanation could be that since the survey focused on folk medicine, respondents took this to imply that the folklorists at the University of Turku were primarily interested in traditional and alternative medical knowledge and practices. Be that as it may, the respondent network nevertheless also included people whose medical knowledge had been formed by health guides composed by medical doctors rather than irregular practitioners.

## Budding health literature

This section provides an overview of the early development and impact of Finnish-language medical and health literature, highlighting key publications and their influence on public health practices and attitudes. Compared with many other European regions and languages, Finnish medical and health literature emerged slowly. For example, by far the most popular health guides in nineteenth-century Europe, Samuel Auguste Tissot’s *Avis au peuple sur sa santé* (1761) and William Buchan’s *Domestic Medicine* (1769), were never translated into Finnish, whereas Swedish editions appeared in 1764 and 1801, respectively.^25^ The first Finnish-language health guides appeared in the 1780s. A minimal, eight-page booklet titled *Konsti elää kauwwan* (1786, 1810; *The Art of Living Long*) was published by an anonymous author in Vaasa.^26^ Christfried Ganander’s *Maamiehen Huone ja Koti Aptheeki* (1788; *The Peasant’s Domestic Pharmacy*) was more significant and had a wider circulation.^27^

The next significant strides in Finnish health literature publishing were taken in the 1830s with the onset of the systematisation of Finnish-language medical terminology. In 1832, Raahe’s district physician Johan Fredrick Ticklén defended a short dissertation titled *Termini medici in lingua Fennica occurrentes.* Ticklén took advantage of Ganander’s publications and Gustaf Renvall’s dictionary of the Finnish language to draw up a Latin-Finnish list of the most common pathological and therapeutic nomenclature.^28^

In the popular health front, however, more influential work was carried out by translators, who provided reading audiences with guides intended for use by the public. Translations of Swedish physician Carl Nordblad’s work, first by Johan Henrik Keckman with the title *Terweyden opetuskirja yhteiselle kansalle* (1837; *The Guidebook of Health for the Common People*), and almost concurrently by Elias Lönnrot as *Suomalaisen talonpojan kotilääkäri* (1838; *Finnish Peasant’s Domestic Physician*), provided advice on self-care and natural medicines. With numerous editions, Lönnrot’s book provided guidelines for many self-learned folk practitioners but was also used in private domestic and communal village settings throughout rural Finland into the early twentieth century.^29^ In a 1916 review of Finnish-language domestic medical books, however, physician and anatomist Yrjö Kajava noted that despite its scientific merits, Lönnrot’s book had become obsolete and that there were many better options available on the book market.^30^

The cultural and social changes brought by urbanisation and industrialisation in the nineteenth century affected people’s perceptions of themselves and the world around them, requiring the acquisition and application of new knowledge.^31^ Altogether, at least two dozen health guides intended for domestic use were published in Finland in the nineteenth century. In addition to books, medical knowledge and hygienic guidelines were spread through newspaper articles and pamphlets.^32^ Larger works were sometimes published as multi-volume booklets, which kept their price affordable.^33^

In addition to Lönnrot’s book, August Timoleon Wistrand’s *Kotilääkäri* (*Domestic Physician*), first published as booklets in the 1890s and then as a book in 1901, was widely distributed and consulted. Wistrand (1807–1866) was a licensed Swedish physician who wrote for both professional and popular audiences. Originally published in Sweden in 1840, his *Handbok i husmedicinen* (*Handbook in Domestic Medicine*; from the seventh edition onwards, titled simply *Husläkaren* or *Handbok*) was in its eighteenth printing by 1889.^34^ It included traditional hygienic guidelines in nutrition, physical exercise and rest, but also detailed descriptions of contagious and noncommunicable diseases as well as a section on commonly used medicaments.^35^ Similar to Lönnrot’s work, Kajava deemed Wistrand’s book outmoded by 1916, but noted that, despite deficiencies and even direct errors in translation, it had been useful in earlier decades. He noted that the guidelines for those who went to fetch a doctor for bedridden patients should be included in every domestic health guide.^36^ Demonstrating how a trusted health manual could remain in use throughout a person’s life, a male respondent (b. 1887) in the 1973 survey referred to Wistrand as a book full of wisdom, still useful after seven decades.^37^

Like many of their European counterparts, Finnish physicians were concerned with what they perceived as degeneration. Medical thinkers regarded it as a threat not only to family and public health, but to the emerging nation as a whole. To counteract the danger, they sought to educate the public via newspaper articles, pamphlets and books. The formative period of Finnish physicians’ hygienic writings was from the 1880s to the Civil War in 1918. Ilpo Helén has argued that interest in public health surged around this time. Health issues and social problems became increasingly interconnected and led to a forceful interest in the development of public health services. Established in 1881, the doctors’ association Duodecim promoted Finnish-language medical practice. Its members were especially concerned with rural health circumstances and actively sought to advance the establishment of the public office of municipal physicians. For these pioneering physicians, the advancement of science was inseparable from nurturing health. Besides academic teaching and research, Duodecim’s activist physicians were also interested in developing public education concerning health care.^38^

In Finland, the fields of hygienic science and social medicine were promoted by esteemed physicians such as August Palmberg, Wilhelm Sucksdorff, and Max af Schultén. These professionals undertook study trips to Central Europe, established professional networks and introduced innovative ideas to Finland, thereby facilitating the country’s alignment with other European nations in terms of hygienic practices. The advancement of hygiene coincided with social medical breakthroughs and the establishment of bacteriology, which was discussed by the Finnish medical establishment early on. Helén has summed up that, for members of Duodecim, hygiene was first and foremost about social hygiene. It was about societal and national health care, primarily aimed at articulating and controlling social problems.^39^

In the late nineteenth century, the Finnish medical society Duodecim had an important role in developing and solidifying the Finnish medical lexicon. Both Duodecim and its Swedish-language counterpart, Finska Läkaresällskapet, published educational booklets and health guides. They also published their own popular periodicals. Duodecim’s journal *Terveydenhoitolehti* (*Health Care Magazine*) was edited by Konrad Relander (ReijoWaara from 1906 onward), and the Swedish-language *Tidskrift för Hälsovård* (*Magazine for Health Care*) by Wilhelm Sucksdorff. They and other hygienists also educated the public through lectures, newspaper articles and pamphlets about various health issues. Initially, *Terveydenhoitolehti* had comparatively high circulation numbers and was widely read in the countryside.^40^ In 1908–1909, ReijoWaara published small booklets in a series titled *Kyläläisten kirjasia* (*Villagers’ Booklets*), which was particularly directed at rural populations. ReijoWaara’s writings in the series included titles on cholera, mother and children, bacteria and disease, intestinal disorders, treatment of wounds, and tuberculosis, among others.^41^

ReijoWaara also edited a section on patient letters in *Terveydenhoitolehti.* Between 1889 and 1916, he received some 2,100 letters from members of the public and answered the majority of them in a column specially dedicated to this purpose. As Anssi Halmesvirta has shown, ReijoWaara listened to the desperate voices of the afflicted and gave regular advice, often suggesting preventative cures. This provided writers from humble backgrounds the opportunity to anonymously express their grievances and intimate concerns, which they may not have felt comfortable discussing in the presence of a doctor. The correspondence reveals how traditional cultural and religious beliefs were interwoven with prevailing medical ideas and concepts to form a sort of hybrid.^42^

A steadily increasing number of social hygiene books were aimed at children, young people and their families, and health education came to cover many areas of life, from advice about breast-feeding to good nutrition and sexual morality for teenagers.^43^ Helén has argued that physicians’ discourse is especially focused on families and homes as the locus of hygienic efforts. Society, nation and each individual had to protect and harden themselves against disease by keeping the body and the environment clean. In this constellation, mothers were in a key position. They gave their children vitality, kept them healthy, and cleaned the home of bacilli and filth. In order to become good mothers, women had to become sensitised hygienically and grow a ‘sense of cleanliness’. In sum, Finnish doctors regarded mothers as their most important allies in national health care.^44^ But the readers did not necessarily experience hygiene in this way, at least not immediately. As Turo-Kimmo Lehtonen has suggested, hygienism’s influence in Finland should not be regarded as linear development but rather as many-sided meandering, a lengthy ‘getting accustomed to’ new ideas.^45^ However, as we suggest further on, hygienic health advice was only one facet of medical popularisation in turn-of-the-century Finland.

## Reception of physician-authored health manuals

The impact and reception of health guides written by Finnish medical doctors varied from acclaim to critical commentary among the Finnish public, as the nature of medical literature and its desired impact on public health practices evolved along with reading practices. Although the efforts to raise the status of Finnish vernacular as an official language and the development of a Finnish literary culture were successful, turning rural populations into active readers was not just an issue of the availability of books. New ideas concerning reading and literacy needed to be cultivated to turn people into active readers.^46^ Educating people to become readers took more than teaching how to read.

The topic of literacy gets little overt coverage in the 1973 survey responses concerning health guides. One responder, after stating that few places had medical books in the early 1900s, speculated further that even if there had been more, they would not have been interpreted properly.^47^ Another voiced a suspicion that they would not have been trusted even if they had been available.^48^ These comments convey the attitude that books would have made little difference for the turn-of-the-century layperson, both on the level of personal literacy as well as in their attitude towards written knowledge. They seem to reflect a stance that the rural populations would not have cared to acquire health guides, even if they were technically available, and if they for some reason did read such books, they would not have used them wisely. In addition, the second comment hints that a higher-educated urban demographic would have known better than to trust them and would have shunned such manuals altogether.

Reinilä, however, has argued that, by the early twentieth century, health guides had spread relatively densely among peasants throughout Finland.^49^ The 1973 survey responses largely confirm this, but it is important not to overgeneralise. Only eighty respondents out of 404 (twenty per cent) gave information specifically on medical books. They also added greater nuances to the chronology of book distribution. One informant wrote that medical books were not nearly as common in earlier periods as they had become by the 1970s.^50^ Another argued that a century earlier, very few medical books were in circulation, but in the twentieth century their number multiplied.^51^

The influence of books in a certain location could have been higher than their low numerical presence in households suggests. In Liminka, northern Ostrobothnia, medical books were in frequent use and circulated among neighbours. The Ruotsinoja ‘manor’ became a centre for book loaning as its owner, a certain Mr. Borg, had allegedly studied medicine. Although he never finished his studies, he earned a reputation as an ‘almost graduated doctor, not a quack’, and people from Liminka and neighbouring communities often sought his assistance for ailments and injuries. A male informant born in 1913 referred to Karolina Eskelin’s booklet *Terveyden hoidosta talonpoikaiskodeissa ja pienten lasten hoidosta* (1899; *Health Care in Peasant Homes and Care of Small Children*) as a work borrowed from the ‘manor’ and frequently consulted by his parents.^52^

Although the nineteenth-century ‘domestic physician’ literature, exemplified by Lönnrot’s and Wistrand’s books, continued to be utilised, it was increasingly supplanted by more scientifically advanced medical books in the early decades of the twentieth century. Moreover, translations of foreign works lost in importance to books composed by Finnish physicians. As Minna Harjula has argued, Finland’s early twentieth-century public health landscape focused heavily on promoting the acceptability of medical knowledge and health services with the intended goal of creating sanitary citizens. As health services were fragmented and centred mainly in southern towns, the hygienic discourse – crystallised in the concept of *kansanvalistus* (enlightenment of the people/nation) – concentrated on preventive measures and health education.^53^ The meagre number of doctors in the Finnish countryside – a situation which only began to change gradually after the independence of Finland in 1917 as a nationwide strategy focused on establishing norms for the municipal supply of services^54^ – must have significantly contributed to the widespread adoption of domestic health manuals.

These ideas were amply in display during the first decades of the twentieth century. Max Oker-Blom’s and Gustaf Väinö Levander’s *Kodin lääkärikirja* (*Household’s Medical Book*), published originally as a series of booklets between 1905 and 1907, was unanimously hailed as the quintessential domestic health guide that sought to ‘make people think about disease in the same way as doctors, namely that disease is not a coincidence or accident, but has its clear causes’.^55^ It was critically reviewed by physician N. J. Arppe in 1907. Although he welcomed the authors’ focus on pathology and medical treatment, he found fault with their prominent fixation on symptoms and diagnostic indications:

I think it is harmful rather than beneficial that, when discussing, e.g. lung disease, they point in detail to pain here and there. It does not help in any way in diagnosis. Instead, listing these kinds of symptoms, which can emerge in connection with several diseases and even when there is no disease, can lead to an increasing number of hypochondriacs. A medical book in the public’s hands is a double-edged sword. A lot of teaching and advice is needed in this field, and therefore, a wisely edited health guide is in place, but even the best medical book can lead to all kinds of misconceptions in less developed readers. Indications read in a book fit perfectly to their symptoms; they have this and that illness, and not only one but several. When they really start experiencing symptoms, they also notice those bodily signs.^56^

Despite these critical remarks, Arppe ended up recommending the book with pleasure because of its good intentions. A decade later, he also published his own domestic health guide, of which more below. Kajava, in turn, hailed the second edition of Oker-Blom’s and Levander’s work, published in two bound volumes in 1911, as the most complete and modern “domestic physician”, drawing especial attention to its profuse illustration.^57^ However, similar critique as laid down by Arppe towards medical books’ utility was fairly common, and there were articles focusing primarily on the potential harm caused by health guides, especially for neurasthenic patients.^58^

After Oker-Blom’s and Levander’s tome, the next highly popular volume was published in 1929, with Karolina Eskelin distinguishing herself as its editor. With over 800 pages, *Kotilieden lääkärikirja* (*Fireside’s* ^59^ *Medical Book*) comprised of popular scientific articles written by medical doctors. It sought to educate the public on natural scientific laws pertaining to health and disease. Eskelin emphasised that real results in advancing public health as well as private well-being could only be gained through cooperation and trust between doctors and patients: ‘There are no miracles nor miracle workers. We must wage our battle by using knowledge and education, not magic and heterodoxy.’^60^

Although *Kotilieden lääkärikirja* focused on medical knowledge and theory, the book also contained a chapter, composed by Eskelin, on the practice of domestic medicine. It was intended as a guideline for those who took care of the sick at home, sometimes for extended periods. Eskelin warned that self-taught caregivers should not think of themselves as real nurses. Nevertheless, as temporary helpers, they could perform valuable service. The book also included a list of medicines needed for a basic home medicine cabinet. In locations closer to a pharmacy, it sufficed to have, for example, camphor, castor oil, aspirin and quinine as internal medicines to treat sudden weakness, laxative disorders, rheumatism, fever and flu. For external use, it was useful to keep a small supply of iodine tincture, calcium liniment, and either a zinc or boron salve. Rural households located far from pharmacists and medical doctors were recommended to keep double supplies of everything needed in a small home medicine cabinet, as well as additional internal and external medicines such as Carlsbad salt, Pyramidon powder, anise liquid, and turpentine oil.^61^

Reprinted several times, a few informants gave credit to *Kotilieden lääkärikirja* and thought it useful. A male informant born in 1900 wrote that the book (1953 edition) contained innumerable good advice and that he used it whenever needed. The book helped him solve smaller issues, but most importantly, by using the book, he knew how to seek further help.^62^ Another male informant (b. 1911), who had worked as a psychiatric nurse, used a popular health guide titled *Suuri lääkärikirja* (*Big Book of Medicine*) to make diagnoses to his friends and acquaintances. He had owned the book since the late 1950s, and jokingly remarked that, had he lived a century earlier, he would have practised ‘the medical profession’ and would have been regarded as ‘a wise man’.^63^

Starting with Oker-Blom’s and Levander’s work in the first decade of the century and continuing with Eskelin’s and others’ popularisation efforts in the 1920s and 1930s, one can see how the importance of practical healing advice diminished over time and was replaced by theoretical, and ultimately encyclopaedic, alphabetically ordered knowledge. This was reflected in several respondents’ answers regarding the latter editions of *Kodin lääkärikirja*, which noted that the book was used for diagnosis but never for advice on curing.^64^ This points to how medicalisation proceeded in Finnish society in the twentieth century. From the medical doctors’ perspective, the purpose of the health guides was to underline physicians’ expert knowledge, and ultimately, to make diseased patients turn to them rather than to quacks. Whereas earlier, nineteenth-century ‘domestic physician’ literature, such as Lönnrot’s *Suomalaisen talonpojan kotilääkäri*, had given ‘peasant intellectuals’ tools to become ‘rational’ folk healers,^65^ this became increasingly difficult if one was to rely only on physician-authored medical books.

As Harjula has argued, the development of health citizenship in Finland can be understood as a series of historical layers rather than linear, successive phases. Elements of earlier ideas and practices were carried on to later structures and institutions.^66^ These layers can also be seen in the reception of medical literature in the 1960s when, according to reviews published in newspapers, Finnish-language medical books continued to enjoy widespread popularity and were greeted enthusiastically, but for different reasons than before. Antti Mattila, PhD in medicine, noted in 1961 that the books intended for a larger audience had to be revised every few years because of the fast advances in medical science and especially in pharmacology. Diagnostics and the course of treatment were to be left to doctors, and the domestic health books became useful only after receiving a diagnosis. Mattila noted that the books were still useful because doctors did not always have time to explain everything to the patient, and sometimes there could be misunderstandings in oral communication.^67^

Similar remarks were made in 1969 with yet another medical book, which explicitly noted that in a longer historical continuum, old health guides almost certainly led readers to observe their bodily symptoms. New medical books, however, had different goals: ‘An enlightened reader wants first and foremost knowledge about oneself, not disease and diseased organs but the normal functioning of one’s body and its structure’. While such books could still provide information about different treatments, they were not methods one could use at home, but technologically advanced procedures followed by physicians.^68^

## Reading sexual health guides

One subgenre of health guides that emerged in the late nineteenth century is the women’s health guide. These have been studied by historian Tiina Männistö, who has explored the production of the female body in Finnish guides aimed at girls and young women between 1890 and 1972. According to Männistö, women’s guidebooks were frequently motivated by a hygienist ethos, aiming to employ health education for the advancement of civility and social purity. Many of them also had religious moral content, although the hygienist movement as such did not always view religion positively.^69^ Notably, these books garnered attention in several responses to the 1973 survey, which are discussed in this section.

In the 1973 survey material, a distinctly women’s health guide was mentioned by three informants. By analysing the different titles recorded on the Finna digital catalogue, it appears they are all referring to the same book by the American author E. B. Duffey, generally titled *Kirja naisille* or *Mitä jokaisen naisen pitää tietämän* (*What Women Should Know about Women: A Book about Women*, originally published in 1879). According to Männistö, the first Finnish vernacular women’s health guide was released in 1890, which would make Duffey an early participant in the market.^70^ The book itself falls into Männistö’s category of young women’s guides, emphasising the importance of health education for civil and moral reasons, and offering medical guidance alongside instructions for social and practical life.^71^

Among the informants, one man mentioned Duffey’s book as part of a longer list of health guides without further elaboration.^72^ On a more interesting note, a woman respondent recalled that she did not receive the book until her sixteenth birthday, implying that she was prevented from reading it until that time.^73^ The last one is a woman who disclosed how her sister, who cared for the house after the death of their mother, had the book. After the informant had her first period, her sister refused to answer her questions seriously and disallowed her from reading the book. The informant proceeded to read it in secret, and says it contained important information which could have been useful beforehand, stating it would have been better to give it to her two years earlier.^74^ Männistö writes that these guides were often meant by authors to be given as gifts on a birthday or other meaningful events, after which she wonders whether or not they were actually used this way or read by the receiver if they were.^75^ Based on this limited evidence, it seems they were at least sometimes given out at specific moments, and young women were interested in reading them.

There are similar stories related to sexual education outside the context of women’s health guides. Männistö references guides aimed at both men and women, as well as those concerned with finding spouses, as closely related to the genre, but considers them outside the scope of her research.^76^ References to these can be found in the survey. A woman remembered having read a book titled *Mitä nuoren miehen ja naisen tulee tietää sukupuoliasioista* (*What a Young Man and a Woman Should Know about Sexual Matters*)^77^ among other books related to sexuality and gender. She also mentioned that they had to be read in secret, although she praised her mother for having informed her about the ‘[…] consequences of surrendering oneself to become the fulfiller of men’s desires before marriage in a very nice and funny way’.^78^ One man mentioned having received a book called *Ohjeita avioliittoon aikoville* (*Instructions for those Intending to Marry*) by ‘a Polish female doctor named Julia Suxdoff [sic]’ and another book called *Aviopuolison valinta* (Choosing a spouse) in 1921. He said they had proven very helpful during his forty years of marriage.^79^ Unrelated to this type of guide but relating to the topic of sexual education, another woman recalled that her mother had removed the pages on sexuality and reproductive organs from the health book in her childhood home. She concluded that ‘[…] in such darkness us children were raised, that even doctors´ books did not inform us of those issues’.^80^

Overall, many of these informants seem to share the opinion that their upbringing concerning sexual matters was needlessly restrictive, and education came too late. Their problem was not a lack of educational books or desire to read them, but a restrictive environment that tried to keep them from doing so. In the survey material, it is women who tend to report being kept from educating themselves. It appears that even if the guides were written for the benefit of civil society, hygienic purity and morality, their subject matter was sometimes considered nonetheless dubious.

## Health fads and practical healing knowledge

The early twentieth century did not lead only to the rise of popular medical books written by Finnish doctors, but also to the proliferation of foreign health fads, ideologies and practices that are currently classified as complementary and alternative medicine. As Nieto-Galan has argued, publications related to alternative practices and their readership grew ‘spectacularly’ despite the increased professionalisation of medical elites in the nineteenth century.^81^ These included practices originating in Central Europe, such as homoeopathy, hydropathy and nature cure, but also North American hybrids such as naturopathy.^82^ Paradoxically, they presented perhaps the most controversial, polemical, and discussed side of popular health literature, but also the most sought-after, for they often promised miracle cures even in ‘hopeless’ cases. These unconventional practices and their impact are the focus of this section.

As Sari Kivistö and H. K. Riikonen have noted, a major discussion raged in Finnish newspapers regarding the reception of Louis Kuhne’s works in the first two decades of the twentieth century. Kuhne was a famous German healer, whose books on nature cure were translated by Anna Kurimo (1869–1938), a pioneer of vegetarianism in Finland. Kuhne’s diagnostic and healing methods were widely discussed and criticised in newspaper articles.^83^ His and other nature curers’ books were generally regarded as humbug, and some practitioners were taken to court for practising medicine illegally.^84^ Despite controversies, nature cure also had devoted followers, and drugless healing gained many long-time adherents, as Suvi Rytty has shown.^85^ A particular overlap between irregular and regular medicine can be detected in water cures, which were part and parcel of not only alternative healing but also formed a significant strand in Konrad ReijoWaara’s medical popularisation, as discussed above. Another physician who recommended water treatments was district physician and tuberculosis-specialist N. J. Arppe, who in 1917 published a popular health guide titled *Kansanlääkäri* (*Folk physician*).^86^

Couéism was another significant health fad that was imported to Finland in the 1920s.^87^ French psychologist and pharmacist Émile Coué’s autosuggestion methods spread globally after his book *Self-Mastery Through Conscious Autosuggestion* was published in England (1920) and in the United States (1922).^88^ In Finland, Couéism became better known via Charles Baudouin’s and Harry Brooks’ work that circulated in Finnish and Swedish translations and led a few aspiring ‘psychotherapists’ to establish ‘Coué Institutes’ in major cities and towns. This caused a major conflict between physicians and self-proclaimed autosuggestive healers. Medical authorities argued that the so-called psychotherapists were illegally practising medicine and brought their concern to the police. In four out of five legal court proceedings, Couéists were condemned as quacks.^89^

The basic mantra-like formula of Couéist autosuggestion was to repeat the phrase ‘Every day, in every way, I’m getting better and better’. This could be modified to address specific bodily ills or mental states and emotions. In the 1973 survey, a male informant (b. 1907) from Hauho, a small municipality located in the Tavastia region, remembered encountering Couéist autosuggestion in a 1925 newspaper that his aunt had sent from America. Suffering from tuberculosis, he had first consulted a medical doctor, who sought to get him a place in a sanatorium. Feeling hopeless and being sceptical about recovering in a sanatorium, the young man, encouraged by his father, who had also been to the United States, decided to try Coué’s method by repeating the basic phrase about twenty times a day. Within a month, he felt fit enough to work again. A couple of years later, in compulsory military service, an X-ray still revealed signs of an old inflammation in the lungs. The informant believed that, in the Winter War, he had saved his feet from freezing in the forty-five-degree cold by using autosuggestion. Shortly prior to responding to the survey, he had used the method to quit cigarette smoking. Coué’s book was reprinted in Finland in 1972, and he bought the book to find relief for emphysema and catarrh through its methods.^90^

**Figure 1.**
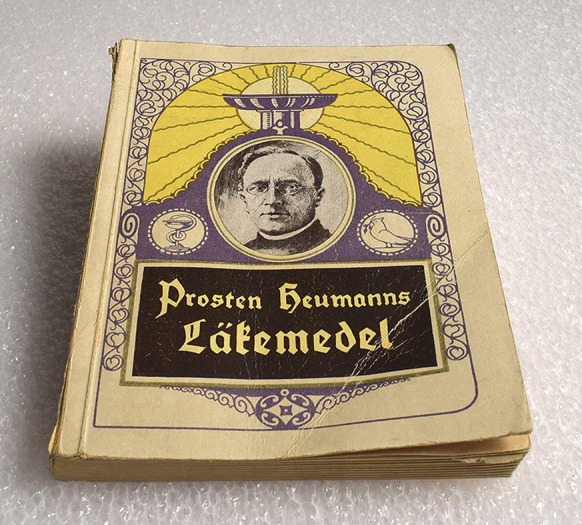
*Prosten Heumanns Läkemedel.* Swedish-language edition of provost Heumann’s patent medicine catalogue/health guide. Photo: City of Turku Museum Center.

Other significant reminiscences in the 1973 survey concern the use of patent medicines and herbal remedies that were marketed via books and booklets.^91^ The most significant among these were the medicines imported from Heumann’s pharmaceutical factory in Nürnberg, Germany.^92^ Their distribution was handled by pharmacist Yrjö W. Jalander, owner of the Leijona (Lion) pharmacy store in Helsinki. In the mid-1920s, he travelled to Nürnberg and set up the distribution network despite strong opposition from other Finnish pharmacists. Because other pharmacies refused to sell Heumann products, Jalander first started advertising them in newspapers and encouraged people to mail order them from the Leijona pharmacy. Together with Swedish associates, he set up a separate company to distribute the remedies and printed a guidebook of Heumann medicines (Figure 1) that exhibited and explained all the separate products and their alleged effects. In his memoirs, Jalander claimed that 350,000 copies of the book were printed and sent without cost throughout Finland. With a population of approximately 3.5 million in 1935, this would have meant that every tenth Finn had direct access to the book.^93^

The marketing of Heumann patent medicines was a great success. According to Jalander, the company had between 150 and 220 daily mail orders of its products, and needed eight employees to deal solely with the packing and sending of medicines. It also started receiving ‘thank you’ letters from the customers. Allegedly, over 9,000 letters arrived within a relatively short time.^94^

While Jalander’s numbers should be taken with caution, it is nevertheless telling that Heumann’s book was the most frequently cited publication in the 1973 survey.^95^ For example, a female informant born in 1906 remembered that ‘c. 30 years ago, Heumann’s medicines were much talked about. They were maybe ordered from somewhere, and they were quite common, and many believed in them.’ She surmised that the Heumann book was the only popular medical publication most people had access to.^96^ One male informant claimed that his brother-in-law, who suffered from liver inflammation and jaundice, healed quite quickly with a Heumann medicine.^97^ However, not everyone shared the excitement. One informant had burned his copy of the Heumann book because, reading it, he concluded that he had almost every disease and disorder mentioned in the book.^98^ Another wrote that, as the Heumann medicines were aggressively marketed with new editions of the book, some people were fooled by the ads and ordered the products, but he did not remember whether they had any noticeable effect.^99^

In addition to the Heumann medicines marketed by Jalander, herbal medicinal products were marketed as miracle cures in interwar Finland. These products were introduced by the Al-Sano cooperative, which was established by a few enterprising Jehovah’s Witnesses in 1931. Al-Sano’s activities focused on publishing and distributing herbal products, especially comfrey (*Symphytum officinale*). The Watchtower movement had spread to Finland in 1909, when Swedish preachers spread Charles Taze Russell’s publications in and around Turku. Watchtower magazine began publication in Finnish in 1912, and in 1921, it was joined by *Kultainen Aika* magazine, which featured translated material from the movement’s *Golden Age* magazine. Significantly, *Golden Age* regularly published health-related articles, including writings on natural medicines.^100^

Al Sano cooperative was led by Karl Henrik Taavitsainen, who had been trained as a masseur and who had been active in the Blind Masseurs’ Association.^101^ Anyone could join the cooperative: between 1932 and 1941, it had between 300 and 400 members. During the Continuation War, the membership rose to over 550 individuals.^102^ The cooperative ran its own drugstore in Helsinki, and another was planned to be established in Tampere. It regularly published a small leaflet, but Taavitsainen made his major impact with a thirty-two-page booklet titled *Kohti terveyttä* (1935; *Towards Health*), which was a collection of articles published in *Kultainen Aika.* The booklet advised people about natural health care. It included testimonials from customers who had tried Al Sano’s products and found them useful. Herbal medicines – including *Symphytum officinale*, *Medicago sativa* and *Ulmus fulva* – were marketed as safe and poisonless. Additionally, Taavitsainen praised the effects of *Lactas calcicus*, olive oil, South American yerba mate, grapes and honey.^103^ Just like the Couéist ‘psychotherapists’ nearly a decade earlier, Taavitsainen and a few central Al Sano employees ended up in court after the booklet’s publication.^104^

Comfrey took pride of place among Al Sano’s products. According to the patient narratives, it had been successfully used to treat asthma, overweight, shortness of breath, jaundice, bone tuberculosis, tumours, and ulcers. Its internal use has now been demonstrated to cause liver toxicity, but many informants in the 1973 survey remembered it as an effective remedy. The most startling (and longest) testimony was given by a male carpenter born in 1907, whose narrative demonstrates the longevity of Al Sano’s ideas and shows that books such as *Kohti terveyttä* could have an impact several decades after their publication:

At least 40 years ago, blackroot [colloquial Finnish word for comfrey], Symphytum Officinale, was a non-prescription product, but for some time now it has been a pharmacy [i.e. prescription] medicine. […] It has become a fashionable medicine which cures everything. A Jehovah’s magazine, Kultainen Aika, advised using it for many diseases, including the curing of lung disease and cancer. I never tried it despite the magazine being distributed for free. For some time, I have been troubled by shortness of breath, and it got worse in 1967, when a biopsy was taken from my lungs to diagnose whether I had cancer. However, what they had seen was a calcified gland outside the lungs. I suspect that the lungs were punctured, and because it was not healed, the cut sometimes opened and let air in the pleura to push the lungs together.

My ailment got worse to the extent that I was granted a disability pension. Penicillin and sulphate tablets helped for a while but did not effect a cure. Then I remembered a small booklet Kohti terveyttä, which I had found in the junk of a torn down house. I knew it was in a box somewhere, had been for over 15 years, and I found it. There was a recipe for preparing blackroot juice. I had seen a similar recipe in a weekly magazine, but that magazine had maybe been recycled. The recipe was: for 1 litre of water, add 40 grams of dried and crushed blackroot. Cook in an iron pot or in an enamel cauldron and let boil carefully under a tight lid. Sieve, let cool under a lid and keep in disinfected bottles in a fridge. In the beginning, take half a cup and then a full cup thrice daily with the meals. Never boil or keep in an aluminium pot, not even in so-called stainless pots, because they often contain aluminium. […] Blackroot is not an instant miracle medicine. I used it for 6 months and am almost healthy. I should have kept on using it, but the pharmacy ran out of it.^105^

Although he dismissed the ‘miraculous’ qualities of comfrey, the informant’s testimony gives an important clue as to why irregular medical advice and alternative health fads became so popular and remained appealing for many decades. Unlike many popular medical books and health guides published by physicians, nature cure and herbal medical guides, as well as patent medicine marketing ploys, gave people something practical and/or tangible to try. Whether bathing or becoming a vegetarian à la Kuhne, repeating a Couéist mantra, or boiling your own comfrey juice, people could get a sense that they were actively doing something to advance their own health. These were often patients suffering from chronic illnesses, who had already consulted medical doctors and tried pharmaceutical medicines.^106^ Therefore, these readers cannot strictly be labelled as ‘non-patients’, as Worboys has suggested.^107^ Psychological and placebo effects were significant when consumers turned to alternative medicines, but it is also noteworthy that the major publications and methods discussed here made grand and unrealistic promises of curing almost any ill imaginable. Responsible medical doctors, of course, had nothing comparable to offer.

## Conclusion

The evolution of popular health guides in Finland from the 1890s to the 1970s reflects a dynamic interplay between medical professionals and the lay public. After slow beginnings, the spread of medical popularisation by print increased in Finland in the final decades of the nineteenth century, making it a latecomer in comparative European perspective. Translations of health guides from other European languages, mainly Swedish and German, provided medical advice until the surge of Finnish- and Swedish-language publications produced by doctors belonging to the Duodecim society or the Finska Läkaresällskapet. Finnish physicians had an important role in propagating hygienic practices, and these guides played a crucial role in disseminating medical knowledge and shaping public health practices, although these new ideas probably took root slowly. The clearest mark of this subgenre can be detected in the written reminiscences that referred to *Kodin lääkärikirja* and *Kotilieden lääkärikirja.*

Three other types of health literature also left a lasting impression on readers. First were the translated publications focusing on sexual and reproductive health. Although female readers seem to have gained more from this subgenre, some men also alluded to it. For women, these books were important in offering knowledge on intimate topics that were usually regarded as shameful secrets by older generations. Second were the guidebooks published by irregular practitioners that were often based on the drugless nature cure and vegetarian practices imported originally from Germany. The Couéist boom in the 1920s can also be regarded as an irregular healing fad. Although the heyday of Couéism was very short-lived, the translated books on the topic could have afterlives of their own. Third, and perhaps most surprisingly, a patent medicine catalogue from the 1930s emerged as the most popular publication in the written reminiscences collected in 1973. Both the nature cure practices and patent medicines shared one thing in common: they promised a cure for almost any disease. These miracle-like qualities form a part of the explanation, but the high-volume circulation of a patent medicine catalogue also demonstrates the effects of aggressive marketing on the medical marketplace.

As noted in the introduction, this article has not sought a close reading of these book-length manuals, but such an approach in a future study could help in bringing more nuance to the transformation in medical knowledge over time, which we have sketched in this contribution. The reception of these guides varied, with some being highly acclaimed for their educational value, while others faced criticism for potentially causing hypochondria. The transition from practical healing advice to more theoretical and encyclopedic knowledge in health guides underscores the increasing medicalisation of Finnish society. This shift highlights the growing emphasis on physicians’ expert knowledge and the importance of turning to medical professionals for accurate diagnoses and treatments. Overall, the study of these health guides and their reception provides insights into the historical development of health literacy and the complex relationship between medical authorities and the general public in Finland.

Readers often had personal and deep relationships with health guides, using them to address specific health issues and improve their well-being. They used medical manuals for practical advice on self-care and natural remedies. Health guides were sometimes shared within communities, with books circulating among neighbours and being used for communal health practices. They were also used for educational purposes, helping readers understand medical theories and practices. Reprints and wide distribution indicate their popularity and impact.

On a final note, we suggest that the worlds of regular and irregular health advice were not as far apart as they sometimes seem to stand in the time of evidence-based medicine, especially in twenty-first-century Finland. Doctors were aware of their low numbers in Finnish society, and for many, this was the main motive for publishing popular health guides. With long distances to the nearest official health care facility, physicians like Konrad ReijoWaara thought it best that domestic medicine should make use of the means available in rural settings, including saunas for cleanliness and woodchopping for building resilience. Irregular healers shared the concern for the patients’ well-being and used some of the same methods advocated by physicians, yet lacked medical training and therefore licences to practice medicine. Nevertheless, via print, their methods found followers or at least people interested in trying them out for often chronic conditions.

## Footnotes

1 University of Turku, The Archives of History, Culture and Arts Studies (hereafter, UTU/AHCAS), Survey 46, Ida Paldanius 10–11. All translations from Finnish to English by the present authors. Here, we have sought a literal translation reflecting the vernacular elements in the Finnish original, including repetition and irregular grammatical structure.

2

V. Vainikainen , Reumatismi ja sydänviat: Neuvoja niiden hoidossa: Muitakin nykyajan luonnonmukaisen terveydenhoidon tärkeitä kysymyksiä (Viipuri: Viipurin kirjatyö, 1930).

3

Suvi Rytty , ‘Puoskarointia vai puhdasta auttamisen halua? Luonnonparantaja Maalin Bergström ja laittoman lääkärintoimen harjoitus 1900-luvun alun Suomessa’, in Markku Hokkanen and Kalle Kananoja (eds.), Kiistellyt tiet terveyteen: Parantamisen monimuotoisuus globaalihistoriassa (Helsinki: Suomalaisen Kirjallisuuden Seura, 2017), 130–64.

4 K.R., ‘Kansanomaista terveyshoitoa’, *Karjala*, 18 October 1930; P.H., ‘Terveydenhoitokirjallisuutta’, *Kansan Lehti*, 10 November 1930; P.H., ‘Terveydenhoitokirja’, *Eteenpäin*, 1 December 1930.

5

V. Vainikainen , Ennenaikaisen vanhuuden ja kuoleman syitä: Luonnollisia elämänohjeita nuorille ja vanhoille (Viipuri: Valo, 1936).

6 On the translated medical literature, see Sari Kivistö and H. K. Riikonen, ‘Lääketieteen ja terveysalan kirjallisuus’, in Outi Paloposki and H. K. Riikonen (eds.), Suomennetun tietokirjallisuuden historia 1800-luvulta 2000-luvulle (Helsinki: Suomalaisen Kirjallisuuden Seura, 2013), 342–64.

7 Although the term was not used in Finnish, we use ‘irregular’ here to denote heterodox – or alternative, in modern terms – medical knowledge and practice that was in variance with official and learned medicine, and was commonly denoted as quackery in nineteenth-to-twentieth-century Finland. On the irregular–regular divide and heterodoxy, see James C. Whorton , Nature Cures: The History of Alternative Medicine in America (Oxford: Oxford University Press, 2002), 8–10 ; Roberta Bivins , ‘Histories of Heterodoxy’, in M. Jackson (ed.), The Oxford Handbook of the History of Medicine (Oxford: Oxford University Press, 2011), 578–97.

8

Suvi Rytty , Ruumiista reformiin: Suomalaiset elämänuudistajat, luonnonmukainen ruumiinmuokkaus ja modernisaation ongelma, 1910–1932 (Turku: Turun yliopiston julkaisuja – Annales universitatis turkuensis, 2021); Kalle Kananoja , ‘Circulating Heterodox Medicine: Globalising and Localising Naturopathy in Interwar Finland’ in H. Weiss (ed.), Locating the Global: Spaces, Networks and Interactions from the Seventeenth to the Twentieth Century (Berlin: DeGruyter Oldenbourg, 2020), 95–116 . On German nature cure practices, see, e.g. John Alexander Williams , Turning to Nature in Germany: Hiking, Nudism, and Conservation, 1900–1940 (Stanford: Stanford University Press, 2007); Avi Sharma , We Lived for the Body: Natural Medicine and Public Health in Imperial Germany (DeKalb: Northern Illinois University Press, 2014).

9 E.g. Osamu Hattori , ‘An Examination of Popular Homeopathic Medical “Manuals” in Germany Published Between the Mid-Nineteenth and Twentieth Centuries’, in O. Hattori (ed.), The Social History of “Manuals” for the Body and Environment: Tools for Education or a Means of Social Control? (Zürich: Lit Verlag, 2017), 105–54.

10

Charles E. Rosenberg , ‘Medical Text and Social Context: Explaining William Buchan’s Domestic Medicine’, Bulletin of the History of Medicine, 57, 1 (1983), 22–42 6344940 ; R. Porter (ed.), Patients and Practitioners: Lay Perceptions of Medicine in Pre-industrial Society (Cambridge: Cambridge University Press, 1985); Ludmilla Jordanova , ‘The Popularization of Medicine: Tissot on Onanism’, Textual Practice, 1, 1 (1987), 68–79 11618210 10.1080/09502368708582008; R. Porter (ed.), The Popularization of Medicine (London: Routledge, 1992); Michael Stolberg , ‘Medical Popularization and the Patient in the Eighteenth Century’, in Willem Blécourt and Cornelie Usborne (eds.), Cultural Approaches to the History of Medicine: Mediating Medicine in Early Modern and Modern Europe (London: Palgrave Macmillan, 2003), 89–107 ; Luzia Savary , ‘Vernacular Eugenics? Santati-Śāstra in Popular Hindi Advisory Literature (1900–1940)’, South Asia: Journal of South Asian Studies, 37, 3 (2014), 381–97; Andrew Schonebaum , Novel Medicine: Healing, Literature, and Popular Knowledge in Early Modern China (Seattle: University of Washington Press, 2016); Jeroen Salman , ‘Print and the Medical Marketplace in the Early Modern Dutch Republic’, Journal for the History of Knowledge, 3, 1 (2022), 1–13 .

11

Roy Porter , ‘Introduction’, in R. Porter (ed.), The Popularization of Medicine 1650–1850 (London: Routledge, 1992), 3 , 9.

12

Roy Porter , ‘The Patient’s View: Doing Medical History From Below’, Theory and Society 14 (1985), 175–9811618212 ; Michael Worboys , ‘The Non-patient’s View’, in Anne Hanley and Jessica Meyer (eds.), Patient Voices in Britain, 1840–1948 (Manchester: Manchester University Press, 2021), 33–60 .

13

Anna-Maria Reinilä , ‘Lääkintä- ja terveyskirjallisuus maallikoiden käsissä’, in Pekka Laaksonen and Ulla Piela (eds.), Kansa parantaa (Helsinki: Suomalaisen Kirjallisuuden Seura, 1983), 130–44.

14

Agustí Nieto-Galan , Science in the Public Sphere: A History of Lay Knowledge and Expertise (Abingdon: Routledge, 2016), 85 .

15

Sofia Kotilainen , Literacy Skills as Local Intangible Capital: The History of a Rural Lending Library c. 1860–1920 (Helsinki: Finnish Literature Society, 2016), 240–2.

16

Laura Stark , ‘The Rise of Finnish-Language Popular Literacy Viewed through Correspondence to Newspapers 1856–70’, in Ann-Catrine Edlund , Lars-Erik Edlund and Susanne Haugen (eds.), Vernacular Literacies: Past, Present and Future (Umeå: Umeå University and Royal Skyttean Society, 2014), 261–77.

17

Martyn Lyons , ‘New Readers in the Nineteenth Century: Women, Children, Workers’, in Guglielmo Cavallo and Roger Chartier (eds.), A History of Reading in the West (Cambridge: Polity Press, 1999), 313–44.

18

Kati Mikkola , Tulevaisuutta vastaan: Uutuuksien vastustus, kansantiedon keruu ja kansakunnan rakentaminen (Helsinki: Finnish Literature Society, 2009), 185–86.

19 Turun yliopisto, Kansatieteen laitos, Tiedusteluja kansankulttuurin alalta, N:o 46, Kansanparantajista ja kansanomaisista parannustavoista. The questionnaire was dated and probably mailed in December 1972, but as the responses were dated in 1973, we have opted to refer to this year throughout the text.

20 Anni Rissanen, ‘Kansanlääkinnän murroskausi: Maailmankuvan muutoksen näkyminen 1900-luvun kansanparantajakokemusten kerronnassa’ (unpublished MA thesis: University of Turku, 2008). To our knowledge, Rissanen’s well-conducted study is the only one that has extensively utilised the 1973 survey. The survey is also referred to in two dissertations, one focusing on the history of cupping in Finland and another on tapeworm extraction in folk medicine. Hindrik Strandberg, ‘Koppning i Finland fram till mitten av 1900-talet’(unpublished PhD thesis: University of Helsinki, 2012); Mervi Naakka-Korhonen , Vaivasta taudiksi: Lapamatoon liittyvä kansanparannus erityisesti pohjoiskarjalaisen aineiston valossa (Helsinki: Finnish Literature Society, 1997).

21 Cf. Kalle Kananoja , ‘Literacy and Healers’ Tactics in Finnish Folk Medicine, 1850–1950’, Social History, 46, 1 (2021), 22–46 .

22 Rissanen, *op. cit.* (note 20).

23 Finnish Literature Society’s Archive (hereafter, FLSA), Competitive collection on folk healing 1978 (Kansanparannuksen kilpakeräys), Aune Aaltonen’s letter, received on 28 October 1977.

24 On thick description, see Clifford Geertz , The Interpretation of Cultures (New York: Basic Books, 1973), 5–10 .

25 On Tissot, see Jordanova, *op. cit.* (note 10); Matthew Ramsey , ‘The Popularization of Medicine in France, 1650–1900’, in R. Porter (ed.), The Popularization of Medicine 1650–1850 (London: Routledge, 1992), 97–133 ; Maria Szlatky, ‘Tissot as Part of the Medical Enlightenment in Hungary’, in *ibid.*, 194–214; Patrick Singy , ‘The Popularization of Medicine in the Eighteenth Century: Writing, Reading, and Rewriting Samuel Auguste Tissot’s *Avis Au Peuple Sur Sa Santé*
’, The Journal of Modern History, 82, 4 (2010), 769–800 . On Buchan, see Rosenberg, *op. cit.* (note 10). Publication years of Swedish translations of Tissot and Buchan have been confirmed from LIBRIS, the online catalogue of the Swedish Royal Library < http://libris.kb.se/>.

26 Kivistö and Riikonen, *op. cit.* (note 6), 343.

27 Reinilä, *op. cit.* (note 13), 134.

28 Kivistö and Riikonen, *op. cit.* (note 6), 345; J. E. Railo , ‘Johan Fredrik Ticklén (1802–1844) ja suomenkielinen lääketieteen sanasto’, Hippokrates: Suomen Lääketieteen Historian Seuran vuosikirja, 3 (1986), 98–123 11638725 .

29 Kananoja, op. cit. (note 21), 38, 40, 42, 44.

30 Yrjö Kajava, ‘Kirjakatsauksia’, *Terveydenhoitolehti*, 28, 2 (1916), 25–29. On Kajava, see Aira Kemiläinen, ‘Kajava, Yrjö’, *National Biography of Finland* online < http://urn.fi/urn:nbn:fi:sks-kbg-006135> (accessed April 1, 2025).

31

Osamu Hattori , ‘Introduction: The Social History of “Manuals” for the Body and Environment’, in O. Hattori (ed.), The Social History of “Manuals” for the Body and Environment: Tools for Education or a Means of Social Control? (Zürich: Lit Verlag, 2017), 7–29 .

32 Otto E. A. Hjelt, *Finlands medicinska bibliografi 1640–1900* (Helsingfors: Finska Läkaresällskapet, 1905), 163–6. Hjelt listed a total of forty-four items (books and articles) from the eighteenth and nineteenth centuries as ‘popular medical texts’, under subheadings ‘popular health guides’, ‘domestic health care, folk texts’, and ‘domestic pharmacies, folk medicines’. Cf. Reinilä, *op. cit.* (note 13), 134, who argues that, by 1880, 15 Finnish-language publications focused restrictedly on medical matters.

33 Kivistö and Riikonen, *op. cit.* (note 6), 342.

34

R. Tigerstedt , ‘Wistrand, August Timolen’, in T. Westrin (ed.), *Nordisk familjebok* 32, (Stockholm: Nordisk Familjeboks Förlags Aktiebolag, 1921), 856–7.

35

A. T. Wistrand , Wistrandin kotilääkäri: Käsikirja kotilääkinnässä: Opetus tuntemaan ja hoitamaan tavallisimpia ulko- ja sisätauteja sekä lyhyt terveys- ja lääkeoppi (Tampere: Kustannus ja kirjapaino osakeyhtiö Sampo), 1901 .

36 Kajava, op. cit. (note 30), 27–28.

37 UTU/AHCAS, Survey 46, P. J. Hänninen 40. However, on an informant not brave enough to use Wistrand’s guidelines for tapeworm extraction, see Naakka-Korhonen, op. cit. (note 20), 260.

38

Ilpo Helén , Äidin elämän politiikka: Naissukupuolisuus, valta ja itsesuhde Suomessa 1880-luvulta 1960-luvulle (Helsinki: Gaudeamus, 1997), 168 .

39
*Ibid.*, 171.

40 In its first year in 1889, *Terveydenhoitolehti* had 7,050 subscribers, but the numbers dropped throughout the 1890s, reaching a nadir of 839 subscribers in 1904 during the editorship (1896–1904) of Fredrik Joel Pätiälä. Relander returned as the editor in 1905 and by 1909 had raised the circulation back to 7,000. In 1913, the magazine had ca. 10,000 subscribers. Jaakko Ignatius, *Suomalainen Lääkäriseura Duodecim 1881–2006*, vol. II (Helsinki: Kustannus Oy Duodecim), 628–31.

41 The series *Kyläläisten kirjasia* was published in Porvoo by WSOY. On ReijoWaara, see Anssi Halmesvirta, *Vaivojensa vangit: Kansa kysyi, lääkärit vastasivat – historiallinen vuoropuhelu 1889–1916* (Jyväskylä: Atena 1998); Pertti Mustajoki, *Mies joka hakkasi halkoja: Terveysapostoli Konrad ReijoWaara* (Helsinki: Duodecim, 2013); Lotta Nikunen, ’”Hyvän hoidon ja tarkan waromisen kautta”: Tartuntatautien esiintyvyys ja ehkäisy Oulun piirilääkärin alueella vuosina 1890–1917 (unpublished MA thesis: University of Oulu, 2024).

42

Anssi Halmesvirta , ‘Despair in Finnish: Consultation by Correspondence in Fin-de-Siécle Finland’, in Tuomas Laine-Frigren , Jari Eilola and Markku Hokkanen (eds.), Encountering Crises of the Mind: Madness, Culture and Society (Leiden: Brill), 139–62.

43

Tuomas Laine-Frigren , ‘Ask the Doctor: Mental Hygiene Among the Young in Fin-de-Siècle Finland’, Social History of Medicine, 37, 2 (2024), 292–311 39323504 10.1093/shm/hkac039PMC11421840.

44 Helén, *op. cit.* (note 38), 172.

45 Turo-Kimmo Lehtonen, ‘Bakteerit ja henkisten ruttotautien siemenet: Puhdas elämä suomalaisessa terveysvalistuksessa 1890-luvulla’, in Timo Joutsivuo and Heikki Mikkeli (eds.), *Terveyden lähteillä: Länsimaisten terveyskäsitysten kulttuurihistoriaa* (Helsinki: Suomen Historiallinen Seura, 1995), 205–52. See also Turo-Kimmo Lehtonen, *Puhtaan elämän jäljillä: Huoli hygieniasta suomalaisissa terveydenhoitolehdissä 1889–1900* (Helsinki: Kuluttajatutkimuskeskus, 1995); Mikko Jauho and Turo-Kimmo Lehtonen , ‘Paha bakteeri, hyvä äiti ja normaaliperhe vuosisadan vaihteen suomalaisessa terveysvalistuksessa’, Tiede & edistys, 23 (1998), 11–25 .

46

Ilkka Mäkinen , ‘From Literacy to Love of Reading: The Fennomanian Ideology of Reading in the 19th Century Finland’, Journal of Social History, 49, 2 (2015), 287–99.

47 UTU/AHCAS, Survey 46, Taimi Pitkämäki 40.

48 UTU/AHCAS, Survey 46, Irja Riiho 53.

49 Reinilä, *op. cit.* (note 13), 136.

50 UTU/AHCAS, Survey 46, Joel Launiainen 13.

51 UTU/AHCAS, Survey 46, Juuso Koivisto 47.

52 UTU/AHCAS, Survey 46, Paavo Kytökorpi 62. August Albert Borg’s (1831–1908) role in providing health care was noted in contemporary newspapers, see, e.g. ‘Kunnianosoitus’, *Louhi*, 12 January 1896. Northern Ostrobothnia did not officially have manors, although some larger estates might have been referred to as such in vernacular expression. We would like to thank Markku Kuorilehto for pointing us to newspaper sources and sharing his information about Ruotsinoja as well as biographical notes about Borg.

53

Minna Harjula , ‘Health Citizenship and Access to Health Services: Finland 1900–2000’, Social History of Medicine, 29, 3 (2016), 573–89.

54
*Ibid.*, 581.

55 N. J. Arppe, ‘Lääkeopillista kirjallisuutta’, *Valvoja*, 27, 10–11 (1907), 674–676.

56
*Ibid.*, 674–675.

57 Kajava, op. cit. (note 30), 28–29.

58

Yrjö Kulovesi , ‘Kansantajuinen lääkeopillinen kirjallisuus ja hermostuneet’, Terveydenhoitolehti, 36, 11 (1924), 192–93; Lauri Taipale , ‘Miten hermostuneisuus ilmenee’, Terveydenhoitolehti, 38, 7–8 (1926), 130–132 . Curiously, these views were echoed in articles by an irregular lay healer. See Sampsa Luonnonmaa, ‘Miten “lääkärikirjat” edistävät sairauksia’, *Terveyden Ystävä*, 2/1930, 36–37, and ‘Lääkärinkirjat edistävät sairauksia’, *Terveyden Ystävä*, 1/1935, 38–39.

59
*Kotiliesi* (*Fireside*), published since December 1922, was one of the first Finnish magazines directed to housewives.

60 Karolina Eskelin, *Kotilieden lääkärikirja* (Porvoo: WSOY, 1929), no page number.

61
*Ibid.*, 762–65.

62 UTU/AHCAS, Survey 46, Juuso Koivisto 47.

63 UTU/AHCAS, Survey 46, Kalervo Haatainen 16.

64 UTU/AHCAS, Survey 46, Saima Airaksinen 51, Onni Lajunen 30, Maria Piri 37, Mauri Röyskö 22.

65 Kananoja, op. cit. (note 21).

66 Harjula, op. cit (note 53), 576.

67 Antti Mattila, ‘Uusi lääkärikirja’, *Uusi Suomi*, 7 May 1961.

68 Matti Leinonen, ‘Lääkärikirja terveesti opiksi’, *Helsingin Sanomat*, 3 March 1969.

69

Tiina Männistö , Haluathan tulla todelliseksi naiseksi? Naisruumiin tuottaminen Suomessa ilmestyneissä nuoren naisen oppaissa 1890–1972 (Turku: Turun yliopisto, 2003), 18–19, 36–38.

70
*Ibid.*, 33. A Swedish translation of Duffey was advertised in *Åbo Tidning*, 14 September 1894. The first one for a Finnish copy is from *Pohjalainen*, 7 September 1897.

71 E. B. Duffey, *Kirja naisille eli mitä jokaisen tytön, morsiamen ja äidin tulee tietää: Naisen kirjoittama naiselle* (Pori: Otto Andersin, 1920), 5. On E. B. Duffey, see, e.g. Cheryl Rose Jacobsen , ‘Lifting the Curse of Eve: Women Writers and Advice Literature on Childbirth’, Women’s Studies in Communication, 18, 2 (1995), 135–51.

72 UTU/AHCAS, Survey 46, Mauno Peippo, 159.

73 UTU/AHCAS, Survey 46, Jenny Koivuniemi 10.

74 UTU/AHCAS, Survey 46, Hilda Kälnäinen 24.

75 Männistö, *op. cit.* (note 69), 29.

76
*Ibid.*, 27.

77 Probably referring to *Mitä nuoren miehen tulee tietää* (What Every Young Man Should Know by Sylvanus Stall; 1905 and several later editions) ja *Mitä tytön tulee tietää* (What a Girl Should Know by Mary Wood-Allen; 1906 and several later editions), published as separate volumes.

78 UTUA/AHCAS, Survey 46, Tilda Salo 20.

79 UTU/AHCAS, Survey 46, Kalle Vilhelmi Niemi 24–25. Niemi probably referred to Julia Sucksdorff, who was married to the above-mentioned hygienist doctor Wilhelm Sucksdorff. Julia Sucksdorff published several books in Swedish, including *Några ord till förlofvade och gifta* (Some Words to the Engaged and Married, 1900). *Aviopuolison valinta* was authored by Vilho Reima, published originally in 1918 and with several later editions. On advice literature intended for men, see Christopher D. O’Shea, ‘Visions of Masculinity: Home-Health Advice Literature, Medical Discourse and Male Sexuality in English-Canada, 1870–1914’ (unpublished PhD thesis: University of Guelph, 2003).

80 UTU/AHCAS, Survey 46, Tyyne Peuralahti 19.

81 Nieto-Galan, op. cit. (note 14), 86.

82

Robert Jütte , Geschichte der Alternativen Medizin: Von der Volksmedizin zu den unkonventionellen Therapien von heute (München: Beck, 1996); Susan E. Cayleff , Nature’s Path: A History of Naturopathic Healing in America (Baltimore: Johns Hopkins University Press, 2016).

83 Kivistö and Riikonen, *op. cit.* (note 6), 354–56. On Kuhne, see also Michael Hau , The Cult of Health and Beauty in Germany: A Social History, 1890-1930 (Chicago: University of Chicago Press), 18–23 .

84 Rytty, *op. cit.* (note 3).

85 Rytty, *op. cit.* (note 8).

86

N. J. Arppe , Kansanlääkäri: Yleistajuisia ohjeita tautien tuntemiseen, parantamiseen ja ehkäisemiseen (Tampere: Kustannus Oy Kirja, 1917).

87

Juhani Ihanus , ‘Couéismi ja “psykoterapeuttiset instituutit” Suomessa 1920-luvulla’, Psykologia 18 (1983), 83–87 .

88 On Couéism, see Dean R. Rapp , ‘”Better and Better–“Couéism as a Psychological Craze of the Twenties in England’, Studies in Popular Culture, 10, 2 (1987), 17–36 ; Eugene Hollahan , ‘”Of Course the Whole Thing Was Couéism”. “The Heart of the Matter” as a Critique of Emile Coué’s Psychotherapy’, Studies in the Novel, 21, 3 (1989), 320–31.

89

Kalle Kananoja , Ihmelääkärit Suomessa 1850–1950: Kuhnekylpyjä, sähköä ja suggestiota (Helsinki: Suomalaisen Kirjallisuuden Seura, 2021), 150–65.

90 UTU/AHCAS, Survey 46, Toivo Armisto 39–41.

91 As Matthew Ramsey has noted for France, a remedy-seller’s catalogue could be disguised as a medicinal manual accessible to anyone. Matthew Ramsey , ‘The popularization of medicine in France, 1650–1900’, in R. Porter (ed.), The Popularization of Medicine 1650–1850, (London: Routledge, 1992), 106 .

92 Heumann was a manufacturer of patent medicines based in Nürnberg and established in 1913. Its catalogue offered products ranging from ‘nerve pills’ to medicines against scabies and haemorrhoids as well as various salves and powders. It also included several veterinary medicines. See *Rovasti Heumannin lääkkeet* (first Finnish edition 1926, several editions between 1928 and 1933); *Heumannin lehti*, 1, 1 (1933), 17. Nowadays, Heumann Pharma manufactures prescription and over-the-counter generic medicines.

93

Y. W. Jalander , 60 vuotta farmaseuttisella alalla (Helsinki: self-published, 1951), 116 . On Jalander‘s controversies with other Finnish pharmacist, see Markku Peltonen , Apteekki suomalaisessa yhteiskunnassa (Helsinki: Suomen Apteekkariyhdistys, 1987), 119 .

94 Jalander, *op. cit.* (note 93), 116. Patent medicines in the Finnish medical market are also discussed in Riitta Oittinen , ‘Health, Horror and Dreams for Sale: Patent Medicine and Quackery in Prewar Finland’, in Robert Jütte , Motzi Eklöf and Marie C. Nelson (eds.), Historical Aspects of Unconventional Medicine: Approaches, Concepts, Case Studies (Sheffield: European Association for the History of Medicine and Health Publications, 2001), 119–38; Petri Paju and Hannu Salmi , ‘Medical and Miraculous Cures on Sale: Newspapers, advertisements and readers between fact and fantasy’, in Patrik Lundell et al. (eds.), Information Flows across the Baltic Sea: Towards a Computational Approach to Media History (Lund: Föreningen Mediehistoriskt arkiv), 183–213 .

95 UTU/AHCAS, Survey 46, Toivo Armisto 27, Paavo Kytökorpi 62, Hulda Koskela 5, Vilho Kolu 42, Eeva Pietilä 6, Viljo Purontaa 27, Helvi Pääkkönen 25, Irja Riiho 53, Hellen Välimäki 33, Helvi Turunen 13.

96 UTU/AHCAS, Survey 46, Hulda Koskela 5.

97 UTU/AHCAS, Survey 46, Toivo Armisto 27.

98 UTU/AHCAS, Survey 46, Paavo Kytökorpi 64.

99 UTU/AHCAS, Survey 46, Armas Karsikas 49.

100

K. H. Taavitsainen (ed.), Kohti terveyttä . Aikakauslehti Kultaisesta Ajasta koottuja kansantajuisia luontaisen terveydenhoidon neuvoja (Helsinki: Al Sano -osuuskunta, 1935).

101

Eero Vartio , ‘Yhdistystoiminnan kehityslinjat ja näkövammaisten tiedonsaannin historia’ in Urpo Kuotola , Anja Tšokkinen and Eero Vartio (eds.), Suomen näkövammaisten ja näkövammaistyön historia (Helsinki: Näkövammaisten Keskusliitto ry, 1988), 257–356 .

102 National Archives of Finland, Helsinki, (hereafter, NAF) Helsingin maistraatin arkisto, Ej 16–27, Osuuskuntien jäsenluettelot liitteineen, Al-Sano-osuuskunta 1932–1943 (Helsinki Magistrate Archives, Member lists of co-operatives, Al-Sano-co-operative 1932–1943).

103 Taavitsainen, *op. cit.* (note 100).

104 NAF, Helsingin raastuvanoikeuden arkisto, Cb IV:287, Helsingin raastuvanoikeuden IV osaston rikosasian asiakirjavihko N:o 114, 1935 (Archives of Helsinki city court, City court’s IV department, Criminal procedures).

105 UTU/AHCAS, Survey 46, Toivo Armisto 28–30.

106 Cf. Whorton, *op. cit.* (note 7), 23.

107 Worboys, *op. cit.* (note 12).

